# A Potential of sFasL in Preventing Gland Injury in Sjogren's Syndrome

**DOI:** 10.1155/2017/5981432

**Published:** 2017-02-23

**Authors:** Jiao Luo, Ying Wang, Bing Yu, Hongyan Qian, Yan He, Guixiu Shi

**Affiliations:** ^1^Department of Rheumatology and Clinical Immunology, The First Hospital of Xiamen University, Xiamen 361003, China; ^2^Xiamen Key Laboratory of Rheumatology and Clinical Immunology, Xiamen, China; ^3^The Chenggong Hospital Affiliated to Xiamen University, Xiamen, Fujian, China

## Abstract

Fas and its ligand FasL, members of tumor necrosis factor receptor superfamily, have been implicated in the process of cell apoptosis. FasL consists of two forms, membrane FasL (mFasL) and soluble FasL (sFasL). sFasL can be produced by mFasL cleaved by matrix metalloproteinases (MMP) and also reveals a role for binding to Fas which is expressed on cell surface. Although Fas/FasL axis has been implicated in a variety of diseases, its role in Sjogren's syndrome still remains ill defined. In this study, we investigated the potential of sFasL in the pathogenesis of Sjogren's syndrome (SS). We found that the serum levels of sFasL in SS patients were significantly lower than healthy subjects. Moreover, serum levels of sFasL in patients with mild disease activity were higher than patients with severe disease activity. There is a positive correlation of the serum level of sFasL with uptake index of parotid gland in our expectation. In addition, liver injury involvement in SS patients showed decreased level of sFasL. Furthermore, we here also observed that the protective cytokine IL-10 expression was positively correlated with sFasL expression. Thus, our results here suggest a potential of sFasL in maintaining gland organ homeostasis.

## 1. Introduction

Sjogren's syndrome (SS) is a chronic autoimmune disease that resulted from immune tolerance breakdown, leading to lymphocytes infiltration in gland organs (salivary gland, lachrymal gland, and the liver) and immune complex deposition as a consequence of B cell hyperactivity [1–3]. The primary manifestation of SS is oral and ocular dryness characterized by lymphocytes infiltration of salivary and lachrymal glands in tissues leading to a progressive destruction of these glands. In addition, liver known as a large secretory organ was also often damaged by the abnormal immune response in SS patients. Autoimmune liver injury accounts for approximately 5%, including primary biliary cirrhosis (PBC) and autoimmune hepatitis (AIH) [4–6].

Fas and its ligand (FasL) are members of tumor necrosis factor receptor superfamily [7]. FasL plays a critical role in the process of cell apoptosis. Human FasL is of 281 amino acids and consists of an 80-amino acid cytoplasmic domain, a 22-amino acid transmembrane domain, and a large extracellular domain. The region of FasL exposed to the outside of the cells consists of 179 amino acids [8]. The extracellular domain is responsible for binding to its receptor Fas. FasL induces apoptotic death of sensitive lymphoid cells expressing its cell surface receptor [9]. Indeed, activated T and B lymphocytes express Fas receptor and thus are sensitive to Fas receptor mediated apoptosis [9, 10]. This has been proposed to be responsible for several regulatory functions of the immune system, including tolerance acquisition, downregulation of immune reactions, and clonal deletion of peripheral lymphocytes [11–14]. Moreover, FasL can be catalyzed by matrix metalloproteinases (MMP) from membranes, which lead to a soluble form sFasL [15]. sFasL could also induce apoptosis of cells when it binds to Fas which is expressed on cell surface. Excessive expression of FasL can inhibit some autoimmune diseases by deleting autoreactive immune cells [16, 17].

Previous findings reveal a role for sFasL in the development of diseases, while the detailed function in the pathogenesis of SS remains unknown clearly. In the present study, we found that serum levels of sFasL were significantly lower in SS patients with mild disease activity, and the levels of sFasL exhibited a positive correlation with uptake index of parotid gland. Furthermore, the SS patients with liver injury showed a decreased level of sFasL. These data suggested that sFasL might exhibit a preventive role in the gland injury in the pathogenesis of SS.

## 2. Subjects and Methods

### 2.1. Patients and Controls

A total of 60 patients diagnosed with SS (57 women and 3 men, age 22–69, mean 47 years) fulfilled the revised version of the European criteria for SS [18]. The patients were recruited from the outpatient clinic and ward of the Department of Rheumatology and Clinical Immunology, the First Hospital of Xiamen University. The results were compared with a population of 20 healthy volunteers (healthy controls) matched for sex and age. Local ethics committee approved the study and informed consent was obtained from patients and control subjects. The number and clinical characteristics of healthy controls and patients with SS were summarized in Table 1. The disease activity is performed by SSDAI score, assessed by constitutional symptoms, change in salivary gland swelling, articular symptoms, hematologic features, pleuropulmonary symptoms, change in vasculitis, active renal involvement, and peripheral neuropathy [19]. Total SSDAI score was 21. Often the disease activity was divided into active and stable state depending on the SSDIA score of 5 points [19, 20].

### 2.2. Detection of Serum Proteins

The protein production of sera from SS patients was determined by Luminex assay from eBioscience (San Diego, CA, USA).

### 2.3. Parotid Gland ECT

43 patients who have Sjogren's syndrome have been injected with 99-mTcO4 through elbow vein and then dynamic salivary gland was scanned. They were orally given vitamin C 0.1 g at the fifteenth minute. Computer produced composite images including dynamic images, time-activity curves, and functional index of all salivary glands were acquired automatically using the technique of region of interest (ROIs) and self-compiled software of the computer. Uptake and excretion index (%) of parotid gland (PG) examined by 99m Tcs was acquired.

### 2.4. Statistical Analysis

All data were analyzed in GraphPad Prism 5. Results are presented as mean ± SEM. The Mann–Whitney *U* test, Spearman's correlation analysis, and unpaired* t*-test with Welch's correction were used to calculate significance. Statistical significance was accepted for *P* values < 0.05.

## 3. Results

### 3.1. Clinical Characteristics of SS Patients

The clinical characteristics of SS patients were summarized for this study (Table 1). Sixty SS patients and twenty healthy control of Southern Chinese population were enrolled in this project. The mean age for SS patients was 47 years with range (23–69), including 57 females and 3 males. Among these 60 patients, there were 2 patients (3.3%) with PBC, 4 patients (6.7%) with AIH, 5 patients (8.3%) with ILD, 2 patients (3.3%) with RTA, and 4 patients (6.7%) with hypothyroidism.

### 3.2. Decreased Serum sFasL Levels in SS Patients

**Figure 1:**
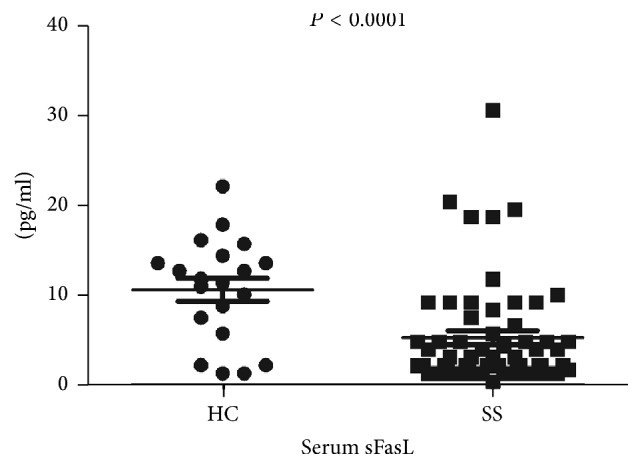
Reduced expression of sFasL in SS patient. The serum levels of sFasL in SS patients (*n* = 60) and healthy controls (HC) (*n* = 20) were detected by Luminex assay. Each symbol represented an individual sample and horizontal lines showed median values. Mann–Whitney *U* test was conducted to compare the data between two groups. *P* < 0.05 was considered as statistic significance.

To explore the role of sera cytokines in the pathogenesis of SS, the Luminex assay was conducted. Compared with healthy controls, significantly decreased serum sFasL levels in SS patients were observed (*P* < 0.0001). As shown in Figure 1, the median level of sFasL in SS patients was 5.352 pg/mL with range of 0.47–30.63 pg/mL, while in healthy controls it was 10.66 pg/mL (1.380–22.16 pg/mL). Although it has been demonstrated that sFasL plays a critical role in many diseases, the role of sFasL in SS patients has not been clearly confirmed.

### 3.3. Relation of Serum sFasL Levels with Disease Activity in SS Patients

**Figure 2:**
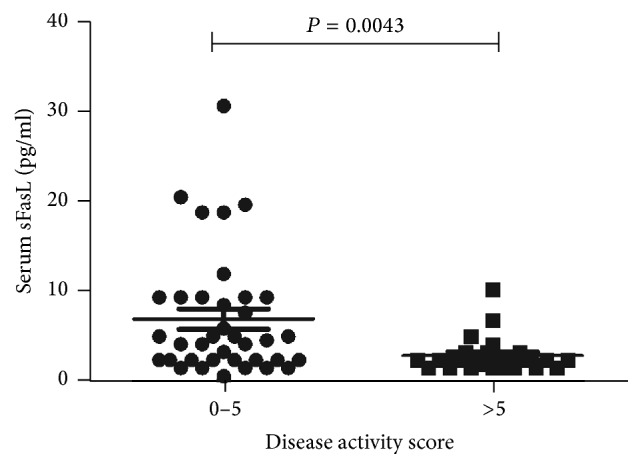
Decreased serum sFasL expression in patients with severe disease activity. The subjects in SS group were grouped into mild (*n* = 37) and severe (*n* = 23) disease activity dependent on the SSDAI score as described in methods. Total SSDAI score was performed when the serum was collected. Each symbol represented an individual sample and horizontal lines showed median values. Mann–Whitney *U* test was conducted to compare the data respectively. *P* value < 0.05 was considered statistically significant.

Regarding the alteration of sFasL in SS patients, the relation between disease activity and sFasL was investigated. The disease activity is performed by SSDAI score, which assessed a combination of clinical history, physical examination, organ specific functional tests, and serologic studies [21]. The SS patients were divided two groups depending on SSDAI score. Here, serum sFasL level in patients with mild disease activity (6.849 ± 1.120 pg/mL) was higher than severe disease activity (2.790 ± 0.4326 pg/mL) (*P* = 0.043) (Figure 2).

### 3.4. Correlation of Serum sFasL Levels with Uptake Index of Parotid Gland in SS Patients

**Figure 3:**
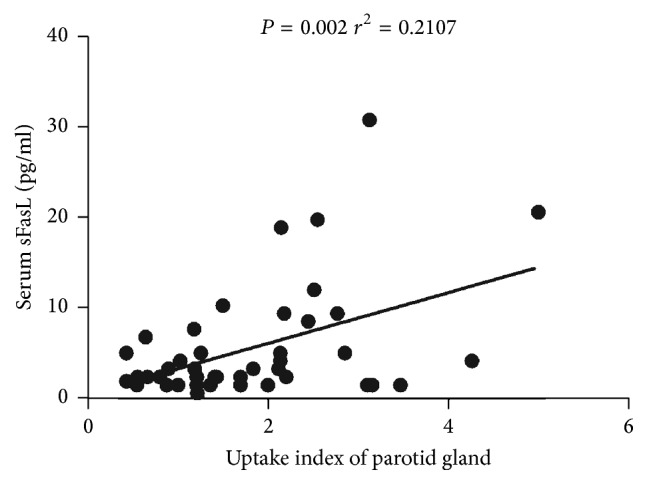
Positive correlation between sFasL and uptake index of parotid gland in SS patients. The determination of linear relationships between sFasL expression and uptake index of parotid gland in SS patients was performed by Spearman correlation coefficient. *P* value < 0.05 was considered statistically significant.

43 subjects of SS patients in this study have taken parotid gland ECT examinations for detecting the gland function. The role of sFasL in the SS patients with parotid gland was explored. As expected, in Figure 3, the sFasL levels were positively correlated with uptake index of parotid gland in the SS patients (*P* = 0.002, *r*^2^ = 0.2107).

### 3.5. Decreased Serum sFasL Expression in SS Patients with AIH

As a secretory organ, liver injury often occurs in SS patients. In our collective SS subjects, 2 SS patients were with PBC and 4 SS patients were with AIH. Furthermore, the sFasL in these SS patients was detected. As expected, in keeping with the parotid gland, the SS patients without PBC/AIH (5.514 ± 0.8254) were higher than SS patients with AIH (2.940 ± 0.7573) (*P* = 0.0338), although there was no significance in SS patients with PBC (5.005 ± 0.8750) (*P* = 0.7467) (Table 2).

### 3.6. Potential of sFasL in Modulating Regulatory Cytokine IL-10 Expression

**Figure 4:**
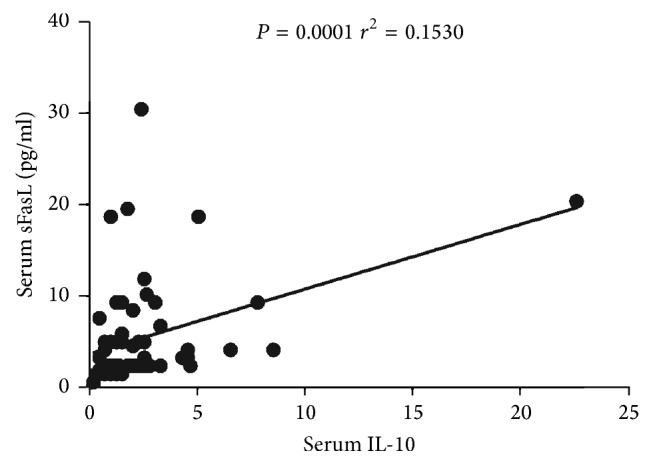
Positive correlation between sFasL with IL-10 in SS patients. The determination of linear relationships between sFasL expression and IL-10 in SS patients (*n* = 60) was performed by Spearman correlation coefficient.

IL-10 is a critical protective cytokine in the development of SS [22]. Thus, the correlation of sFasL and IL-10 was analyzed. Interestingly, we demonstrated that there is a positive correlation between sFasL and IL-10 (*P* < 0.0001, *r*^2^ = 0.1530) (Figure 4).

## 4. Discussion

In our study, we demonstrated the sFasL expression and its potential role in SS patients. Serum sFasL levels were significantly reduced in SS patients when compared with healthy control subjects. Furthermore, serum sFasL levels of patients in stable state were higher than patients in active state. In addition, we also found that the sFasL levels were positively correlated with uptake index of parotid gland in the SS patients and a decreased expression in SS patients with liver injury. Interestingly, a positive correlation between sFasL and IL-10 was investigated. These data suggested that sFasL might prevent damage of gland and plays a protective role in the pathogenesis of SS.

The natural course of the disease and the onset of salivary gland dysfunction were not clearly known [23]. Previous report has found that serum sFas and sFas-L levels were significantly higher in some rheumatic disease patients [24]. In children with chronic kidney disease (CKD), sFas/sFasL ratio may be a new marker of inflammation and endothelial dysfunction [25]. However, the role Fas/FasL axis in SS which refers to keratoconjunctivitis sicca and xerostomia due to lymphocytic infiltrates of lachrymal and salivary glands remains unknown [26]. The glandular infiltration in SS is composed mainly of CD4+ T lymphocytes [27] and also contains a substantial number of B cells and plasma cells [28, 29]. The infiltration of lymphocytes into glandular aggregates apparently plays a crucial role in the tissue pathology of SS [30]. Apoptosis of activated T lymphocytes is essential in the regulation and timely resolution of inflammatory and immune responses [31]. It was evident that the interaction of Fas with FasL regulates a large number of pathophysiological processes of apoptosis including autoimmune diseases [32]. Accumulated evidences suggest an important role of apoptosis in the pathogenesis of SS [33]. Apoptosis of salivary glands T cells was decreased in SS patients [34]. It was demonstrated that sFasL treatment has potential therapeutic benefit in reducing inflammatory infiltrate and neovascularization in primary and recurrent forms of herpetic stromal keratitis and that it does so by augmenting the restriction of Fas+ inflammatory cells mediated by membrane FasL [35]. Previous study also provided evidence that sFasL could be related to the mechanism involved in the elimination of the parasite [36]. Some study also found that sFasL might mediate the apoptosis of T lymphocytes [37]. However, a study showed that levels of sFasL were significantly increased in SS patients [24], which is contrary to our results here. The difference might be resulting from the various race, age, treatment, and disease duration time of subjects. In our study, sFasL was found decreased in SS patients and the sFasL levels were positively correlated with uptake index of parotid gland in the SS patients, and the SS patients with PBS/AIH showed a decreased expression of sFasL in sera. These data showed that sFasL may prevent damage of gland injury from active lymphocytes through inducing apoptosis.

Imbalance of cytokines also was implicated in the damage of gland in SS patients. It has been suggested that T helper type 1 (Th1) cytokines, such as IFN-*γ*, IL-2, and IL-6, might be important in the induction and/or maintenance of SS [38]. Recently, activated T cells remain resistant to Fas stimulation and their sensitivity to apoptosis increases gradually, regulated by a number of interrelated factors, including availability of FasL-presenting cells and the pro- and antiapoptotic factors and the presence of cytokines [11, 39–41]. Previous study demonstrated that the Breg cells and Th1, Th17, and Th22 cells showed a negative correlation, and IL-10, IFN-*γ*, IL-17, and IL-22 levels expressed by these cells also showed a negative correlation [42]. These activities are mediated, at least in part, by the production of IL10 and may control a variety of autoinflammatory diseases including inflammatory arthritis, inflammatory bowel disease, autoimmune diabetes, SLE, and SS [43]. Inflammatory cytokines (IL-2, IL-5, IL-6, and IL-12) tend to protect Th1 cells from apoptosis, while immunomodulatory/anti-inflammatory ones like IL-10 have a proapoptotic effect [31, 44–46]. In our study, a positive correlation of sFasL with IL-10 was observed. sFasL can promote apoptosis of immune cell resulting in preventing parotid gland injury in Sjogren's syndrome. At the same time, we also found that sFasL might have a protective role in the damage of liver in SS patients with AIH.

In conclusion, the results presented here suggest that the serum sFasL may play a beneficial role in the pathogenesis of SS gland injury. These data maybe suggest a novel approach in the SS treatment. Certainly, further studies are required to explore the specific regulation mechanism between sFasL and SS.

## Figures and Tables

**Table 1:** Demographic data and clinical characteristics of subjects in the study.

Characteristics	SS patients	Healthy control
Total	60	20
Age at study mean (SD) years	47	46
Female sex	57 (95%)	19 (95%)
PBC (primary biliary cirrhosis)	2 (3.3%)	—
AIH (autoimmune hepatitis)	4 (6.7%)	—
ILD (interstitial lung disease)	5 (8.3%)	—
RTA (renal tubular acidosis)	2 (3.3%)	—
Hypothyroidism	4 (6.7%)	—

**Table 2:** Serum sFasL levels in liver damage.

Group	Number (*n*)	Serum sFasL (pg/mL)	*P* value
SS patients without PBC/AIH	54	5.514 ± 0.8254	—
SS patients with PBC	2	5.005 ± 0.8750	0.7467
SS patients with AIH	4	2.940 ± 0.7573	0.038^*∗*^

^*∗*^ *P* values were obtained from the statistical comparisons of serum sFasL levels among the various study groups. SS patients without PBC/AIH were compared with SS patients with PBC and with AIH. Unpaired *t*-test with Welch's correction was used. Statistical significance was accepted for *P* values < 0.05.
